# Climato-Economic Origins of Variations in Uniqueness of Nickname on Sina Weibo

**DOI:** 10.3389/fpsyg.2021.599750

**Published:** 2021-05-13

**Authors:** Lingnan He, Yue Chen, Xiaopeng Ren

**Affiliations:** ^1^School of Communication and Design, Sun Yat-sen University, Guangzhou, China; ^2^Institute of Psychology, Chinese Academy of Sciences, Beijing, China

**Keywords:** uniqueness, climate demand, social ecology, social media network, China

## Abstract

In the world of social media, people are free to choose names based on their preferences, which may potentially reflect certain levels of uniqueness. In this study, we have attempted to explore the possibility of applying the ecological theory of individualism/collectivism in the context of social media. We, thus, examined provincial variations in the uniqueness of nicknames among more than 13 million Sina Weibo users. Initially, the nickname uniqueness indicator was set at the provincial level. It was found that the uniqueness of nicknames was the highest in provinces with temperate climates, for example Guangdong, and the lowest in provinces with demanding climate, such as Ningxia. Regression analysis results partially supported that inhabitants in provinces with temperate climate were more likely to use unique nicknames on social media compared to those from harsh climate. This finding is significant in terms of ecology.

## Introduction

Billions of people all over the world currently have their profiles in online social networks (OSNs). OSNs are widely used as the primary medium for communication and networking. Users often use nicknames, rather than their real names, as identifiers to be found and remembered by others, as well as to express their preference. Cultural studies on real names found that the uniqueness of real names at the group level is an indicator of individualism and functions as a social–ecological factor (Twenge et al., 2010; Varnum and Kitayama, 2011).

Does it also work for nicknames? Some authors have argued that people on online social media platforms behave in a similar manner to real settings and function as if it is real life (Back et al., 2010). This suggests that inhabitants in an individualistic culture or region would be inclined to pick unique nicknames for themselves than their counterparts in a collectivistic one. However, there is little research on the subject.

Measurable variations of collectivism have been proven within Mainland China (Hou et al., 2016; Stojcic et al., 2020). Some socio-ecological factors were found to predict provincial variations of collectivism, such as climate demand (Van de Vliert et al., 2013) and herder–wheat–rice farming (Talhelm et al., 2014; Stojcic et al., 2020). Sina Weibo has 516 million users all over China (Statista, 2020). Millions of users’ nicknames could be used to measure their uniqueness. Therefore, we tried to examine whether there are provincial differences in the uniqueness of nicknames and its relationship with climate demand.

## Uniqueness

Uniqueness is a key facet of individualism/collectivism (I-C) (Oyserman et al., 2002). I-C may be the most important dimension that captures cultural differences in human behavior (Oyserman, 2017). Compared to an individualistic culture, people in a collectivistic one are more embedded in their tight social networks and care more about relational or group harmony. I-C has been proven to be a loosely connected construct, in which the different facets or measurements are weakly related to each other (Na et al., 2020). People in an individualistic culture prefer to differ from others or stand out from groups than those in a collectivistic one. In a content analysis of 27 individualism/collectivism scales, personal uniqueness was identified as one of seven facets of individualism, in which about 30% of the scales included items measuring personal uniqueness, such as “I’m different from others in many respects.” Furthermore, in the follow-up meta-analysis, uniqueness was found to be sensitive to cultural differences (Oyserman et al., 2002). Kim and Markus found that European Americans would select pens with unique colors as presents as opposed to Asian Americans, as the differently colored pens indicated their uniqueness (Kim and Markus, 1999). When naming babies, people are more inclined to use unique names in an individualistic culture or regions than those in a collectivistic culture. As a result, the percentage of the most common first names has been constructed as an indicator of I-C, where a higher percentage of the most common first name means higher collectivism or less individualism for a certain group. For example, a study that used data from 13 nations, which included nine European nations and four frontier nations (United States, Canada, Australia, and New Zealand), found that the percentage of the most common first names was negatively correlated to Hofstede’s individualism (Varnum and Kitayama, 2011). Furthermore, along with the increase in individualism in the United States, people had started giving babies unique names, which resulted in a decrease in the percentage of the most common first names (Twenge et al., 2010).

The link between uniqueness and I-C exists not only in intercultural comparisons but also in intracultural regional comparisons. Furthermore, intracultural comparisons would give an additional validity for controlling the more confounding factors such as language, religion, etc. (Liu et al., 2019). In Varnum’s studies 1 and 2, the percentages of the most common baby names were higher in less individualistic regions (eastern regions) than in the more individualistic ones (western frontier regions) in both the United States and Canada separately (Varnum and Kitayama, 2011). Others found that frontier experiences during 1790–1890 were associated with giving babies uncommon names at the county level in the United States (Bazzi et al., 2020). Overall, fitting in or standing out from a group in the practice of naming babies has been strongly proven to be a facet of I-C.

## Climato-Economic Hypothesis of Uniqueness

In general, uniqueness is an outcome of psycho-behavioral adaptation toward a climate demand and the income resource of inhabitants’ environment. Derived from the demands and resources theory, the climato-economic theory of culture posits that culture is shared psycho-behavioral adaptations toward the interaction of climate demands and economic resources (Van de Vliert, 2013, pp. 467–469). Since a temperate climatic condition is undemanding and comforting, its inhabitants do not need much resources to survive; thus they make easygoing goals a priority over survival goals. They then like to adopt a convenient agency to deal with problems. They would neither feel pressure to follow the group norms and fit in nor have a strong motivation to stand out from a group when picking up a nickname. Compared with those in regions with moderate climates, inhabitants in regions with extreme summers and harsh winters need more resources to sustain life, such as housing, clothes, and food, in order to cope with the climate stress. Whether inhabitants appraise climate demand to be either threatening or challenging is moderated by the existing resources to cope with it. In hotter-than- and colder-than-temperate climate conditions with poor resources, inhabitants would evaluate it as a threatening condition, which makes them prioritize survival goals over easier goals. They then would prefer to adopt an in-group agency to deal with problems. This implies that inhabitants would be prone to sacrifice some individual freedom to obtain support and resources from groups in order to survive. They are sensitive to group norms and are inclined to conform rather than to stand out in order to maintain group harmony. As a result, inhabitants would be risk-averse and like to pick a common nickname that fits in the group rather than a unique one that stands out. When in hotter-than- and colder-than-temperate climate conditions with rich resources, inhabitants would deem it as a challenging condition and prioritize self-expression over others. Furthermore, they would be likely to adopt an individualistic agency to deal with problems. They would be motivated to be confident and prefer to be self-reliant rather than be dependent on groups for resources to survive. Consequently, inhabitants would be likely to pick a unique name that probably stands out from the group and fits their self-expression goals and the norm of “not following the social norms” rather than picking a common nickname that fits in the group and the norm of “following the social norms.”

Although there is no direct evidence of a relationship between climato-economic conditions and the uniqueness of naming practice, there is empirical evidence that supports the climato-economic hypothesis of I-C. For example, using World Value Survey data, it was found that members of societies in more demanding climate patterns endorse survival values at the expense of self-expression to the extent that the household incomes in these lower-income societies are lower (Van de Vliert, 2007). The interaction of climate demand and income resources also predicted compatriotism, nepotism, and familism (Van de Vliert, 2011), freedoms (Van de Vliert, 2013), overall subjective ill-being composed of health complaints and anxiety and burnout (Fischer and Van de Vliert, 2011), and in-group/out-group differentiation (Van de Vliert, 2020). Thus, it can be suggested that the climato-economic theory of collectivism could predict uniqueness in naming practice.

## Username on Online Social Media

Numerous individuals across the world have their personal profiles on social media, where they engage in different kinds of activities. Usernames, instead of their real names, are often used as labels or markers to be easily found by other individuals or organizations. Usernames are also a convenient way to express the owner’s preferences, attitudes, emotions, and so on, especially so for the Chinese as they have a long history of assigning certain connotations or implications to names. Furthermore, unlike a real name, a nickname is usually given by the user himself/herself rather than by his/her parents, which means that the nickname is more closely linked to the individualistic or independent value of the user compared to the real name. Thus, the most common username at group level can be used as an indicator of I-C, following the same mechanism as mentioned before.

## China: A Natural Case

China has an advantage in exploring the relationship between climate demand and the uniqueness of usernames. Firstly, provincial variations of I-C have been found in Mainland China, such as nepotism, thinking style, implicit individualism (Talhelm et al., 2014), composited collectivism (Van de Vliert et al., 2013), and cultural tightness–looseness (Chua et al., 2019). Secondly, ecological factors differ from each other to the extent that they are similar to the national differences in Europe (Ren et al., 2021). For example, the climate demand in Heilongjiang (91.3), which is located in northern China, is similar to that in Norway (89), while those in Hainan (22.9), which is located in southern China, is similar to that in Cook Island (28) (Van de Vliert, 2013; Van de Vliert et al., 2013). Thirdly, the interaction of climate demand and income resource could predict collectivism at the provincial level within the Chinese mainland using a sample from 15 provinces (Van de Vliert et al., 2013). Lastly, the uniqueness of a name, which is made up of the percentage of the most common or popular names (reversed), has been found to support I-C ethnic or regional differences within Mainland China and their relationship with ecological factors such as herding vs. farming (Stojcic et al., 2020) and voluntary frontier settlement (Chen et al., 2019).

The shortcomings of China should also be addressed to explore the effects of climato-economic factors. Firstly, most Chinese provinces have demanding climate conditions (Van de Vliert et al., 2013). Secondly, China is an underdeveloped nation with poor resources from a global perspective. For instance, China’s per capita gross domestic product (GDP) ranked 70th worldwide in 2019 (World Bank, 2020). Thus, the overall livability in China would be appraised as threatening rather than challenging (Van de Vliert et al., 2013). Variations of climato-economics would not be large enough so that their effects on uniqueness are mild or medium.

**FIGURE 1:**
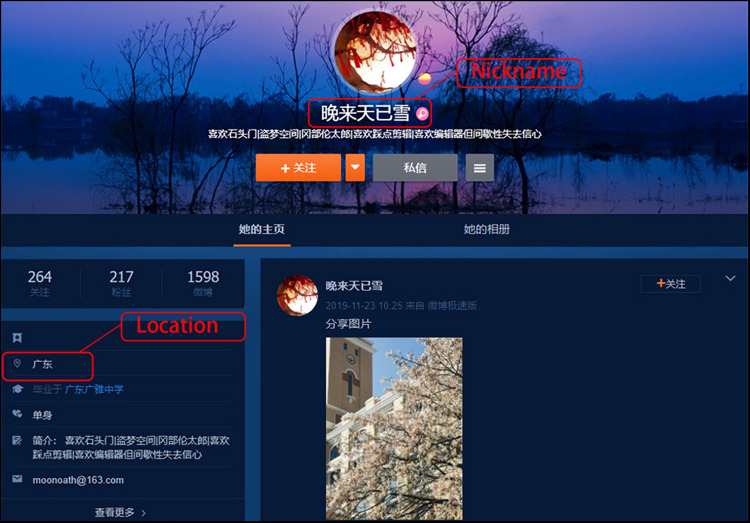
Web page of a Sina Weibo user with the nickname and location.

Sina Weibo, often referred to as Chinese Twitter, is one of the most influential social networking platforms in China. Over 350 million users are registered on Sina Weibo. Each day, around 130 million words and 1.5 million videos and live streaming are published on the platform (Statista, 2020). Nicknames and location information of registered users could be found publicly on their home page. This is illustrated by a screenshot of a user from this platform (see Figure 1), with a nickname “wan lai tian yi xue” (in Chinese, “晚来天已雪”) and located in Guangdong. They gave us a chance to explore whether or not there are regional variances of name preferences and its relationship with climate demand.

Unfortunately, the percentages of the most common names that had been used before do not work for the usernames on the Sina Weibo platform. When one username such as “renxiaopeng” was used, a new user cannot use this username again on the platform. He/she would be forced to create a new username. It is impossible to use the previous method to calculate the percentages of the most common usernames. Thus, we strived to find a new method to construct the uniqueness of usernames so that regional differences of uniqueness within China can be measured.

Therefore, the aims of the current study were to: (1) create an index of uniqueness based on Sina Weibo users’ nicknames at the provincial level and (2) test whether or not the climato-economic theory of collectivism could predict the uniqueness of nicknames at the provincial level in China. We hypothesized that the uniqueness of nicknames would be lowest in provinces with a harsh climate and lower incomes, moderate in those with temperate climate irrespective of income, and highest in those with a harsh climate and higher incomes.

### Ethics Statement

This study involving human participants was reviewed and approved by the Institutional Review Board of the Institute of Psychology, Chinese Academy of Sciences. Written informed consent from the participants was not required to participate in this study, in accordance with the national legislation and the institutional requirements, because our data were collected from publicly available information on an open online social networking site through Weibo’s application programming interface.

## Part 1: Creating the Chinese Uniqueness Scale

### Methods

Following Vandello and Cohen’s and Yamawaki’s strategy in creating a collectivistic scale (Vandello and Cohen, 1999; Yamawaki, 2012), we created the Chinese uniqueness scale at the provincial level (similar to states in the United States and prefectures in Japan) using Sina Weibo users’ nicknames. Based on characters of the users’ nicknames, we selected a few indicators that we thought were theoretically related to uniqueness. Thereafter, individual items would be moderately correlated to yield a scale with high reliability. These items would have to be non-redundant to fit the parsimonious principle. Finally, a few items were deleted, which will be discussed later. Thus, three items were retained on the Chinese uniqueness scale.

Generally, a user’s nickname is quite different from his/her real name. Each Chinese real name is composed of a surname and a first name, in which the surname is written before the first name, for example, Ren Xiaopeng (in Chinese, “任孝鹏”). The first name usually includes one or two Chinese characters. Non-Chinese characters, such as an Arabic number and an English alphabet, are excluded. However, all these rules are set aside for nicknames. A nickname with far more than three characters or with English alphabets and Arabic numbers can be found easily. This deviation from the rules of real names in China implied constructing uniqueness based on nicknames.

#### Percentage of Users With Over 10 Chinese Characters in Their Nickname

As mentioned in the paragraph above, real names mostly comprise two or three Chinese characters, for example, Ren Xiaopeng (in Chinese, “任孝鹏”) or Su Hong (in Chinese, “苏红”). For a nickname, it is also common for users to use four to seven Chinese characters to create a nickname to express his/her uniqueness, for example, “na dou bu shi shi” (in Chinese, “那都不是事”). However, compared with common nicknames, those with over 10 items are infrequent, such as “meng meng xiao jie shi ge ao jiao de xiao gu liang” (in Chinese, “萌萌小姐是个傲娇的小菇凉”). Ten Chinese characters can be considered to be more unique than most nicknames. Thus, having a nickname with over 10 Chinese characters was used as the inclusion criterion for this study.

#### Percentage of Users With Non-chinese Characters in Their Nickname

Non-Chinese characters include English alphabets or Arabic numbers or other symbols, such as “^∗^.” For example, the nickname “NorthToFace” was thought to be unique.

#### Percentage of Users With Nicknames With No More Than Five Full Chinese Characters (Reversed)

Nicknames with all Chinese characters and no more than five full Chinese characters will be similar to a real name, for example, “qing jing ben ran” (in Chinese, “清净本然”). It sounds reasonable to be a common nickname. Therefore, it was reversed to make it unique.

### Results

A total of 16 million Sina Weibo users’ posts were randomly extracted from January 1 to December 31, 2017 through Weibo’s application programming interface. Users without location information were excluded. Over 13 million users’ nicknames from Mainland China were selected for analysis. Users were unevenly distributed all over Mainland China. There are 1,682,967 users in Guangdong Province, while only 39,718 users in Tibet. This are listed in Table 1. All three indicators were calculated on the users’ nicknames.

**TABLE 1:** Provincial ranking on Chinese uniqueness scale index.

Rank	Province	Score	Nswu	Rank	Province	Score	Nswu
1	Ningxia	28	62456	17	Yunnan	45	221916
2	Tibet	31	39718	18	Heilongjiang	46	266395
3	Qinghai	32	48843	19	Zhejiang	48	758421
4	Jiangxi	36	243245	20	Jiangsu	50	941354
5	Anhui	37	379311	21	Guizhou	52	143428
6	Hebei	38	443475	22	Shaanxi	52	351092
7	Henan	38	601851	23	Inner Mongolia	54	185780
8	Shandong	40	759389	24	Hubei	55	466695
9	Hainan	42	91762	25	Sichuan	55	598154
10	Fujian	43	459472	26	Chongqing	55	287059
11	Guangxi	43	250784	27	Tianjin	57	369552
12	Liaoning	43	432508	28	Beijing	59	1119240
13	Gansu	44	137517	29	Shanghai	94	779648
14	Jilin	44	205736	30	Guangdong	95	1682967
15	Hunan	45	355395	31	Xinjiang	102	161522
16	Shanxi	45	261719				

*Nswu, number of Sina weibo’s users.*

We calculated the indicators of uniqueness using the following procedures. Firstly, the raw scores of three indicators of uniqueness at the provincial level were derived as a function of the number of those with the above-mentioned characteristics divided by the number of users. Secondly, the scores for each item were standardized across 31 provinces. Thirdly, the overall uniqueness of the nickname score was the sum of individual (*Z*) scores for the three items. Finally, we transformed the scores by multiplying the means by 20 and then adding 50, to ensure a uniqueness score ranging from approximately 1 to 100. On this new uniqueness of nickname scale, a higher score represents a higher uniqueness.

As shown in Table 2, the overall standardized alpha for the three-item index was 0.845. For nicknames with over 10 characters and nicknames with non-Chinese characters, the deletion of these two items would decrease the reliability. But the deletion of nicknames with no more than five Chinese characters or with full Chinese characters would increase the reliability. Furthermore, we included this item for two reasons: (1) more than two items should be included to ensure the reliability of the scale and (2) it is important to expand the scope of uniqueness by nickname and only minimally impact the overall alpha. As for the corrected item total correlations, all items were above 0.400, recommended by other similar indicator index scales (Yamawaki, 2012). In addition, all the intercorrelations among these three items were moderately and positively correlated as desired, from 0.560 to 0.745 (Table 3). Overall, the three-item index met our statistical criteria of acceptability.

**TABLE 2:** Reliability statistics for three uniqueness indicators.

	Corrected item-total correlations	Alpha if item deleted
PNOT	0.727	0.540
PNE	0.667	0.614
PNFC(R)	0.638	0.852

*PNOT, percentage of nickname over 10 characters; PNE, percentage of nickname with non-Chinese character; PNFC, percentage of nickname with full Chinese character lower than 5. R indicates the item is reverse scored. Total standardized α = 0.845.*

**TABLE 3:** Correlation matrix for three uniqueness indicators.

	PNOT	PNE	PNFC
PNOT	–		
PNE	0.745**	–	
PNFC(R)	0.629**	0.560**	–

PNOT, percentage of nickname over 10 characters; PNE, percentage of nickname with non-Chinese character; PNFC, percentage of nickname with full Chinese character lower than 5. R indicates the item is reverse scored. ^∗^*p* < 0.05; ^∗∗^*p* < 0.01; ^***^p < 0.001.

## Part 2: Relationship of Uniqueness With Climate

Having established the reliability and face validity of the uniqueness scale, we then used it to test the main hypothesis of uniqueness and the climato-economic factor in Mainland China. Other factors, such as population density and the percentage of people farming rice/wheat, could also be controlled for.

Climate demand was calculated as the sum of the four absolute deviations from 22°C for the average lowest and highest temperatures of the provincial capital in January and July following the method of Van de Vliert et al. (2013).

Income resource was measured by GDP per capita for each province in 2017.

### Control Variables

#### Urbanization

The total and urban populations of 2017 were taken from the sixth population census (conducted in 2010)^1^. Urbanization was derived by dividing the urban population by the total population.

#### Percentage of Populations With College Degree

The population with college degree of 2017 was taken from the sixth population census (conducted in 2010) (see text footnote 1). The percentage of the population with a college degree was derived by dividing the population with a college degree by the total population.

#### Population Density

Population density was taken from the Yearbook of Urban–Rural Development 2017^2^.

#### Percentage of Minorities

Ethnic population data were taken from the sixth population census (conducted in 2010) (see text footnote 1). The percentage of minorities was derived by dividing all non-Han ethnic population by the total population.

#### Percentage of Herding Minorities

Following Talhelm et al. (2014), a few minorities such as the Mongolian Chinese were included as herding minorities. The percentage of minorities was derived by dividing all herding ethnic population by the total population.

#### Percentage of Cultivated Land Devoted to Rice Paddies

The data for this variable were extracted from Talhelm et al. (2014).

### Results

We performed all analyses in SPSS 25.0. Xinjiang, Inner Mongolia, Tibet, and Qinghai were excluded for their herding traditions. As Table 4 shows, zero-order correlations between the independent and dependent variables showed that the uniqueness index was positively related to income resource (*r* = 0.549), urbanization (*r* = 0.615), percentage of the population with college degree (*r* = 0.446), and population density (*r* = 0.665) and was negatively correlated to climate demand (*r* = −0.278, n.s.). Although climate demand did not correlate with the uniqueness index significantly, it was also put into the next-regression analysis given the relatively small size (*N* = 27), following Vandello and Cohen’s procedure.

**TABLE 4:** Correlation matrix for uniqueness index and other variables.

	PCS	Urban	PD	PM	PHM	PR	IR	CD	Unique
PCS									
Urban	0.841**								
PD	0.656**	0.736**							
PM	–0.232	−0.397*	–0.285						
PHM	0.141	0.149	–0.096	0.061					
PR	–0.100	0.133	0.266	–0.002	–0.346				
IR	0.821**	0.958**	0.751**	−0.412*	0.096	0.095			
CD	0.201	0.152	–0.066	–0.204	0.477*	−0.636**	0.116		
Unique	0.446*	0.615**	0.665**	–0.252	–0.118	0.355	0.549**	–0.278	

PCS, percentage of population with college degree; Urban, urbanization; PD, population density; IR, GDP per capita; CD, climate demand; PM, percentage of minorities; PHM, percentage of herding minorities; PR, percentage of paddy rice; Unique, uniqueness indicator; *p < 0.05, **p < 0.01; *N* = 27.

We ran a hierarchical regression analysis with the uniqueness index as a dependent variable to explore the main and the interaction effects of climate demand and income resource following Van de Vliert’s method. As Table 5 shows, the results in the first step indicates that the population density (β = 0.445, *p* = 0.071) and urbanization (β = 1.733, *p* = 0.009) had an effect on uniqueness, while the percentage of paddy rice had no effect on uniqueness. In the second step, climate demand (β = −0.425, *p* = 0.06) and income resource (β = −0.954, *p* = 0.088) had a marginally significant effect on uniqueness. The interaction of climate demand and income resource had a marginally significant effect on uniqueness (β = −2.629, *p* = 0.074), whereas the main effects of climate demand (β = 0.573, *p* = 0.321) and income resource (β = 1.525, *p* = 0.289) were insignificant. However, it was suggested that the interaction effect be excluded due to collinearity issues (variance inflation factor, VIF = 55.5).

**TABLE 5:** Hierarchical regression predicting uniqueness at provincial level.

	Model I	Model II	Model III
PCS	–0.314	–0.236	0.095
Urban	1.733**	1.437*	1.060^¥^
PD	0.445^¥^	0.470^¥^	0.321
PM	–0.081	–0.081	0.040
PHM	–0.012	–0.012	0.114
PR	–0.168	–0.168	–0.224
IR		−0.954^¥^	1.525
CD		−0.425^¥^	0.573
IR × CD			−2.629*

PCS, percentage of population with college degree; Urban, urbanization; PD, population density; IR, GDP per capita; CD, climate demand; PM, percentage of minorities; PHM, percentage of herding minorities; PR, percentage of paddy rice; ^¥^*P* < 0.10, *p < 0.05, **p < 0.01; *N* = 27.

Finally, we ran a regression analysis with the uniqueness index as the dependent variable to explore the main effects of climate demand and income resource. The results showed that climate demand (β = −0.347, *p* = 0.036; β = −0.425, *p* = 0.060) and income resource (β = 0.589, *p* = 0.001; β = −0.954, *p* = 0.088) have an effect on the uniqueness of nicknames if other control variables were excluded.

## Discussion and Conclusion

We developed a three-item index, named Chinese uniqueness of nickname index, designed to measure the degree of uniqueness across the provinces of China. Two pieces of evidence were provided to test its validity. Since the three items have face validity, it was obvious that nicknames with over 10 Chinese characters would be considered as more unique than those with three to five Chinese characters. For example, the nickname “meng meng xiao jie shi ge ao jiao de xiao gu liang” (in Chinese, “萌萌小姐是个傲娇的小菇凉”) would be more unique than “wan lai tian yi xue” (in Chinese, “晚来天已雪”). Modernity indexes such as income resource and urbanization were positively related to the uniqueness index at the provincial level.

Our results also lent partial support to the climato-economic theory of collectivism. Harsh climates may encourage inhabitants to favor group harmony and avoid being different from group members to cope with bad environment (Van de Vliert, 2013). When people from these areas choose names for themselves on social media, they prefer to use common names, which results in a lower uniqueness at the provincial level. Although livability has improved with the advent of modernization, climate demand remains imprinted on the inhabitants’ variations on uniqueness.

Income resource was not supported in this study. At first glance, income resource predicted uniqueness as more income resource meant that the inhabitants were more likely to select a unique nickname on social media. However, this is in contrast to the previous finding in the Chinese mainland. The previous study found that income resource would moderate the effect of climate demand on collectivism as inhabitants of those areas with harsh climatic demand and higher income resource would be least collectivistic (Van de Vliert et al., 2013). This implies that such inhabitants would select a more unique nickname. Some factors may explain this inconsistency. Firstly, income resource is the secondary factor in the climato-economic theory of collectivism, in which the interaction with climate demand was more important than its main effect (Van de Vliert, 2013, p. 507). Unfortunately, due to the small size of the provinces and collinearity, the interaction effect could not be used to support the climato-economic theory. Secondly, the main effect of income resource on collectivism is not robust. Even in their study in China, the main effect of income resource was not always significant on collectivism (Van de Vliert et al., 2013; Table 4). Thirdly, income resource was just one of the resources that could be used to cope with hot or cold climate stress. Other resources such as geographical resources could be explored in future studies.

Our findings make theoretical contributions to both the climato-economic theory of culture and computational social psychology. Firstly, it enriches the climato-economic theory of collectivism by exploring its effect on real behavior in OSNs. In this series of studies on the climato-economic theory of collectivism, Van de Vliert relied heavily on secondary data and the self-report measure of collectivism (Van de Vliert, 2007, 2011, 2013). While such rating scales are good at assessing individual differences within a group, they should be complemented by other measures such as cultural products or cognitive tasks (Chen and Kitayama, 2013). Our results found that climate demand could at least predict the uniqueness in nickname preference. Inhabitants in provinces with harsh climatic demands are less likely to make up unique nicknames than those from provinces with comfortable climate demands. This result highlighted its generalizability not only on psychological traits but also on behavior in real settings.

The study also has contribution to computational social psychology. Big data presents unprecedented opportunities to understand human behavior on a large scale. Data and computer scientists often use bottom-up, data-driven methods to predict social psychological variables, such as personality traits, by Facebook likes (Youyou et al., 2015) or just algorithms from machine learning based on the digital records of users (Kosinski et al., 2013). This makes it difficult to elucidate without other evidence. There is a possible way to solve this problem of big data as including theoretically relevant variables as predictors could complement any shortcoming of the data-driven method (Qiu et al., 2018). Our study attempted to combine a data-driven explanation with a theoretical one. Firstly, we conceptualized the uniqueness index theoretically and then found that climate demand could explain provincial variations on uniqueness. It helped to provide a meaningful explanation of social psychological phenomena with big data and avoid mere predictions (Qiu et al., 2018).

Our findings also lend support to the advantage of intranational-level unit analysis in the link between ecology and cultural values (Vandello and Cohen, 1999). Compared with national-level unit analysis, intranational-level unit analysis (such as provinces in China or states in the United States) within one culture or nation reduces criticisms based on language, religions, measurement errors for large nations, sociological indicators of collectivism, and psychometric equivalence across distinct populations (Van de Vliert et al., 2013). In our study, other confounding factors such as language and religion were well controlled for. The overwhelming majority of Sina Weibo users would be Han Chinese. Increasingly, studies have combined national and intranational comparisons to explore the relationship between cultural values and ecological factors (Thomson et al., 2018).

China is a natural and ideal cultural entity to explore the relationship between cultural values and ecological factors. On the one hand, from the geographical and ecological perspectives, the range within China is similar to that in Europe and the United States. Until now, subsistence-style work such as herding and farming rice/wheat had been proven using a Chinese sample (Chua et al., 2019; Stojcic et al., 2020). On the other hand, since China has been keeping records and maintaining databases for over 2,000 years, this could be used to explore the origin of cultural values with spatiotemporal evidence in the future.

Our results did not support the rice theory of collectivism. Farming rice/wheat has been found in the Chinese mainland to predict that inhabitants in rice-farming provinces would be more likely to be collectivistic than those in wheat-farming provinces (Talhelm et al., 2014; Dong et al., 2019). Our results, however, showed that the percentage of paddy rice did not predict uniqueness. We put aside the significance of the coefficient that Sina Weibo users from rice-farming provinces were more likely to give themselves unique nicknames than those from provinces farming wheat, which is indicative of the opposite of the collectivistic theory.

Collectivism is a loosely connected concept in which different facets may be sensitive to different ecological factors. For instance, in-group favoritism and out-group exclusion may be more sensitive than holistic thinking to the perceived threat of infectious diseases (Fincher et al., 2008). In this study, uniqueness may be sensitive toward the climate demand, but not to farming rice/wheat.

Population density was found to predict uniqueness positively: inhabitants in provinces with a higher population density prefer more unique nicknames than those in lower ones. This was consistent with the hypothesis that a higher population density is associated with a stronger collectivism (Vandello and Cohen, 1999; Gelfand et al., 2011). Within China, population density was highly correlated with the modernity indicators such as urbanization and GDP per capita. Modernity predicted uniqueness positively. The effect of population density on uniqueness probably was reverted or offset by modernity.

We also considered some limitations. Firstly, some authors have argued that intra-provincial variances should not be neglected, especially the relationship between ecological factors and collectivism (Ren et al., 2021). We avoided it because not enough ecological data were available at the city or the county level. It could be improved in the future to explore this topic at the city level, when available. Secondly, three factors were included in this study. Other factors such as infectious diseases could also be explored to make the results robust. Thirdly, location labels reported by the users themselves were thought to be the actual provinces where they grew up. It was impossible to guarantee the accuracy of the classification. However, we do not believe that this would pose a significant problem. As most people report their actual location on social network platforms, we are open to new methods and research to find a better way to obtain users’ locations in order to explore whether or not our finding is robust.

Overall, based on Sina Weibo users’ nicknames, we constructed a uniqueness index with reliability and face validity. Furthermore, the study also found that climate demand could explain and predict uniqueness. Harsh climate could make inhabitants unlikely to use unique nicknames on online social platforms.

## Data Availability Statement

The raw data supporting the conclusions of this article will be made available by the authors, without undue reservation.

## Ethics Statement

This study involving human participants were reviewed and approved by the Institutional Review Board of the Institute of Psychology, Chinese Academy of Sciences. Written informed consent from the participants was not required to participate in this study in accordance with the national legislation and the institutional requirements because our data was collected from publicly available information on an open online social networking site through Weibo’s application programming interface.

## Author Contributions

XR: conception and design of the study. LH and YC: acquisition of the data. XR and LH: analysis and interpretation of the data and drafting the manuscript. XR, LH, and YC: revising the manuscript critically for important intellectual content. All authors contributed to the article and approved the submitted version.

## Conflict of Interest

The authors declare that the research was conducted in the absence of any commercial or financial relationships that could be construed as a potential conflict of interest.

## Footnotes

1 www.stats.gov.cn

2 www.mohurd.gov.cn
